# A rare case of cutaneous melioidosis manifesting as infective panniculitis: a case report

**DOI:** 10.1186/s12879-025-10939-x

**Published:** 2025-04-18

**Authors:** Tanat Yongpisarn, Pitak Santanirand, Suwichak Chairanaicharoen, Teerapong Rattananukrom

**Affiliations:** 1 Division of Dermatology, Department of Medicine, Faculty of Medicine, Ramathibodi Hospital, Mahidol University, 270 Rama VI Road, Ratchathewi, Bangkok, 10400 Thailand; 2 Clinical Pathology, Faculty of Medicine, Ramathibodi Hospital, Mahidol University, Bangkok, Thailand

**Keywords:** *Burkholderia pseudomallei*, Infection-induced panniculitis, Melioidosis, Case report

## Abstract

**Background:**

Melioidosis, caused by the gram-negative bacterium *Burkholderia pseudomallei*, is a heterogeneous disease with diverse clinical manifestations, including cutaneous involvement in 10–20% of cases.

**Case presentation:**

We report the first documented case of infection-induced panniculitis caused by direct inoculation of *B. pseudomallei*. A 32-year-old woman from Mukdahan, Thailand, with chronic myeloid leukemia treated with a tyrosine kinase inhibitor, was hospitalized for allogenic stem cell transplantation. Three days before admission, she developed a solitary erythematous papule on her left forearm, which rapidly progressed to a tender subcutaneous nodule following chemotherapy. Her hobby of caring for cacti exposed her to soil and caused repeated cactus pricks on her upper extremities. Incisional biopsy of the lesion revealed mixed lobular and septal neutrophilic panniculitis with ischemic fat necrosis, without evidence of vascular occlusion or vasculitis. *B. pseudomallei* was isolated from a tissue aerobic culture, while blood cultures were negative. She was diagnosed with cutaneous melioidosis. After intensive treatment, the lesion healed, leaving a hyperpigmented patch and a biopsy scar.

**Conclusion:**

This case highlights the importance of considering melioidosis in patients presenting with panniculitis, particularly those with immunosuppression, minor trauma, and exposure in endemic regions.

**Supplementary Information:**

The online version contains supplementary material available at 10.1186/s12879-025-10939-x.

## Background

Melioidosis is a heterogeneous disease with a wide range of clinical manifestations, including cutaneous involvement in approximately 10–20% of cases [1]. It is caused by *Burkholderia pseudomallei*, a facultative intracellular gram-negative bacterium, commonly found in soil and surface groundwater [1, 2]. While historically endemic to Southeast Asia and northern Australia, melioidosis has increasingly gained attention in other regions such as Africa and the Americas [1, 2]. 

Cutaneous melioidosis is most frequently manifested as a nonhealing solitary ulcer at the site of inoculation, typically on the lower leg [1]. Other cutaneous manifestations include abscesses, cellulitis, pustules, boils, and carbuncles [1]. Rarely, melioidosis has been reported to present with atypical dermatological manifestations, such as Sweet syndrome [3] and polyarteritis nodosa [4]. However, to the best of our knowledge, infective panniculitis caused by direct inoculation of *B. pseudomallei* has not been previously reported. In this report, we present the first case of infection-induced panniculitis caused by direct inoculation of *B. pseudomallei*.

## Case presentation

The patient is a 32-year-old non-diabetic female who lives in Mukdahan, Thailand, where she works as a teacher, and she engages in cactus breeding as a hobby. In April 2022, she was diagnosed with chronic myeloid leukemia (CML) and has been treated with a tyrosine kinase inhibitor (TKI). Initially, she received imatinib for nine months before switching to ponatinib in February 2023 due to a lack of cytogenetic response. In November 2024, she was hospitalized for allogenic stem cell transplantation (ASCT) following unsuccessful TKI therapy. Her baseline white blood cell (WBC) count was 5250 /µL (neutrophils 43.7%, lymphocyte 40.9%, monocyte 13.6%, basophil 1.8%).

**Fig. 1:**
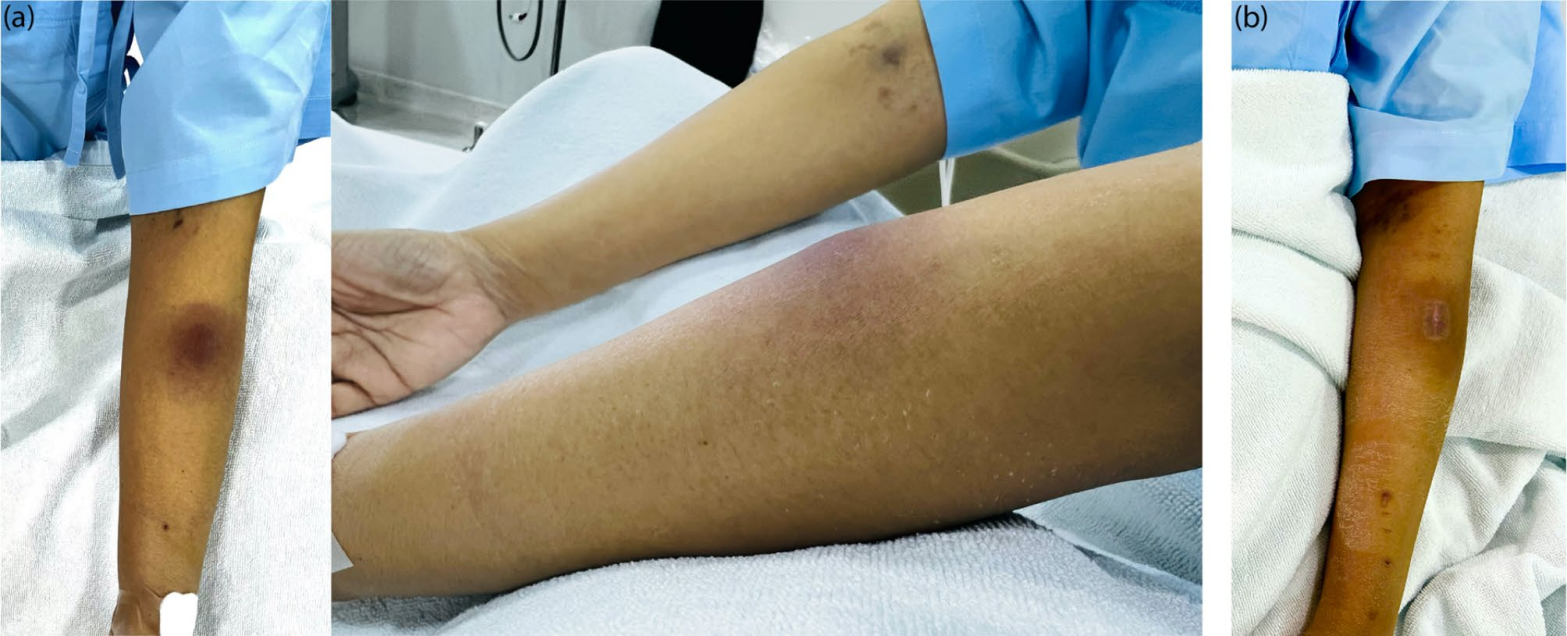
Illustrates the clinical presentation of the patient at the time of biopsy, which was characterized by an erythematous subcutaneous nodule on her left forearm (**a**) and the resolution of the lesion appearing as a hyperpigmented patch with a biopsy scar (**b**)

A solitary erythematous papule on her left forearm had developed three days prior to admission. She was given chemotherapy with fludarabine and busulfan as part of the conditioning regimen. Four days later, her skin lesion grew into an erythematous subcutaneous nodule, measuring 2 cm in diameter, tender on palpation (Fig. 1a). Additionally, she developed leukopenia (WBC, 1270 /µL) and a fever of 39.1 ^o^C, prompting empirical cefepime therapy and a septic work-up. One day later, she developed pancytopenia with febrile neutropenia, and her laboratory tests revealed leukopenia (WBC, 290 /µL), mild anemia (hemoglobin, 8.4 g/dL), and thrombocytopenia (platelet, 20,000 /µL). She subsequently proceeded with the stem cell transplant.

**Fig. 2:**
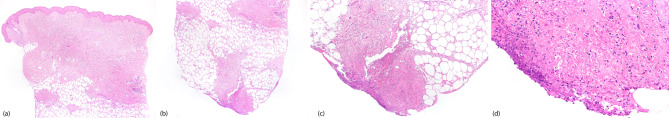
demonstrates the histopathological findings of the patient, which consist of mixed lobular and septal panniculitis (**a** and **b**, hematoxylin–eosin (H&E); ×40), dense diffuse cell infiltration (**c**, H&E; ×100), neutrophilic infiltration with nuclear dust, and fat necrosis (**d**, H&E; ×400)

**Fig. 3:**
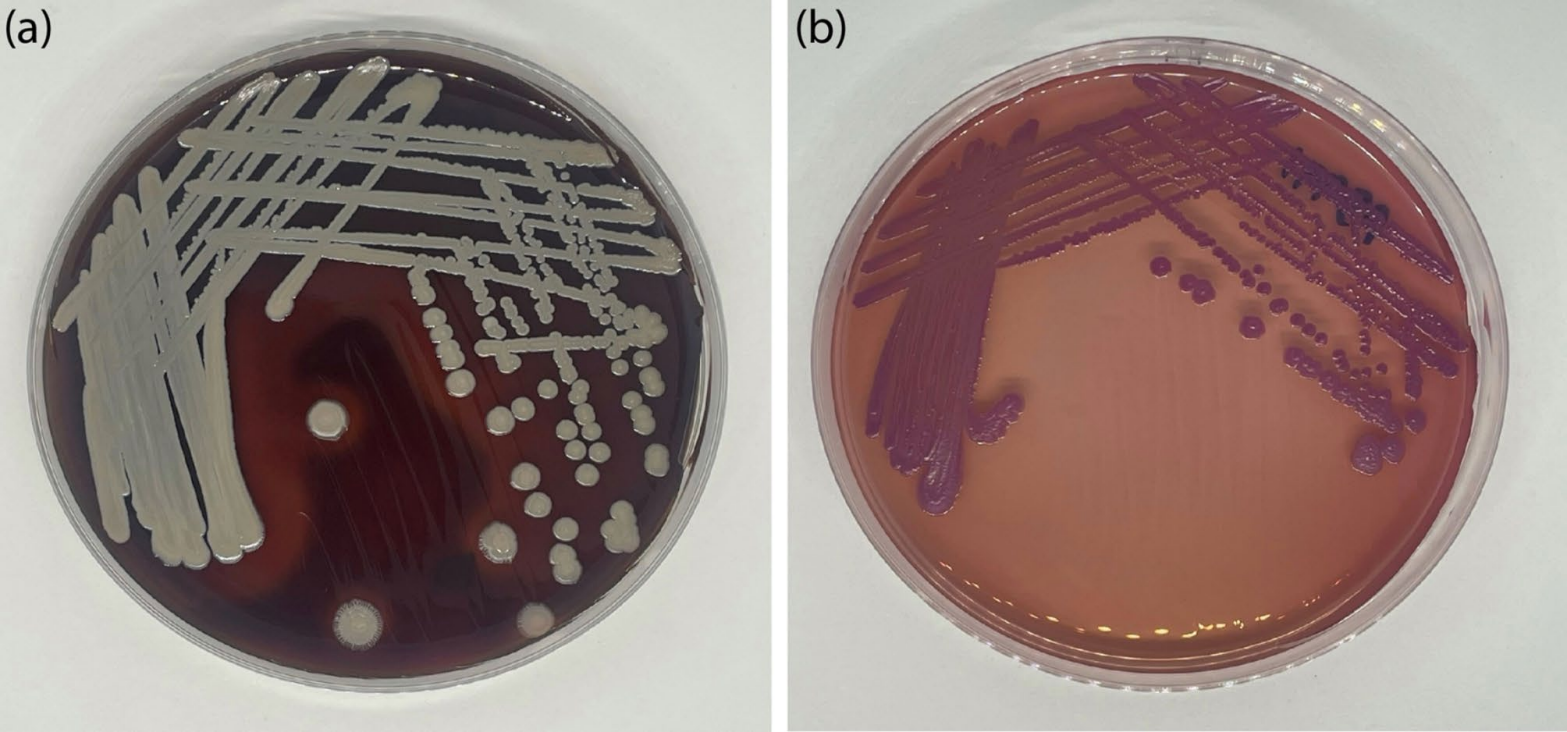
*Burkholderia pseudomallei* growth on day 5 at 37 ^o^C on blood agar (**a**) and the characteristic dry, wrinkled, pinkish-purple colonies with a metallic sheen on the MacConkey agar (**b**)

A dermatology consultation was requested two days following ASCT. An incisional biopsy was consequently performed on the left forearm. Histopathology revealed mixed lobular and septal panniculitis, as well as neutrophilic infiltration with nuclear dust, and ischemic fat necrosis, without evidence of vascular occlusion or vasculitis (Fig. 2). These findings were consistent with neutrophilic panniculitis, which requires additional investigation to determine an infectious etiology. Although special stains, including Brown-Brenn, Fite, acid-fast bacilli, Gomori methenamine silver, and periodic acid-Schiff, did not reveal any organisms, *B. pseudomallei* was subsequently isolated from a tissue aerobic culture three days later. The organism was identified by conventional methods, including colony morphology, Gram stain, and biochemical tests. Small, smooth colonies of *B*. *pseudomallei* appeared on day 2, which then underwent gradual changes to form the characteristic dry, wrinkled, pinkish-purple colonies with a metallic sheen on the MacConkey agar on day 5 (Fig. 3). In addition, the MALDI-TOF MS (Bruker Daltonics, Bremen, Germany) was used to confirm the species. The protein profiles of 16sRNA sequencing confirmed *B. pseudomallei* strains were added as an in-house reference library. The antimicrobial susceptibility testing was performed using a customized THAN4F panel (ThermoFisher, USA) by the micro-broth dilution technique. The result interpretation was performed according to the CLSI M45, 3rd edition guideline [5]. The strain was susceptible to ceftazidime (minimum inhibitory concentration or MIC, 2 µg/mL), imipenem (MIC, ≤ 0.5 µg/mL), amoxicillin-clavulanate (MIC, ≤ 4 µg/mL), and trimethoprim–sulfamethoxazole (MIC, 2 µg/mL).

She underwent a computerized tomography scan to investigate the possibility of extra-cutaneous involvement; however, no pulmonary consolidation or intraabdominal abscess was detected. Hemocultures did not identify any organism, and she was subsequently diagnosed with cutaneous melioidosis, in addition to her preexisting conditions, including febrile neutropenia in a post-ASCT host.

Prior to the diagnosis, she had been receiving a broad-spectrum antibiotic regimen, including meropenem, vancomycin, amikacin, and voriconazole. During the intensive phase of her cutaneous melioidosis treatment, she was prescribed meropenem 1 gram (25 mg/kg/dose, up to 1 gram) every 8 h for a total of 19 days, followed by de-escalation to ceftazidime 2 g (50 mg/kg/dose, up to 2 g) every 8 h for 4 days. Following the intensive phase of treatment, her skin lesion healed after 3 weeks, leaving a hyperpigmented patch with a biopsy scar (Fig. 1b). The eradication phase was concluded with oral trimethoprim–sulfamethoxazole (240/1200 mg) every 12 h (8/40 mg/kg/day) for at least 3 months.

## Discussion

Based on the patient’s history and clinical presentation, the differential diagnosis for panniculitis included infective panniculitis [6], subcutaneous Sweet syndrome (SS) [7], and erythema nodosum (EN) and leukemia cutis. It is difficult to differentiate between each form of panniculitis by clinical alone, but infective panniculitis was favored due to the acute onset, immunocompromised status, and association with sepsis [6]. EN is the most prevalent form of panniculitis and can develop in response to a variety of antigens, such as infection, inflammation, drug use, and malignancy [8]. Although EN often involves lower extremities, lesions on the upper extremities appear to be associated with hematologic malignancy [8], as seen in our patient. In addition, both subcutaneous SS and leukemia cutis can also be associated with her underlying hematologic malignancy; [7] although leukemia cutis was less likely due to its typically more gradual onset.

The histopathological findings of lobular and neutrophilic panniculitis can be found in both infective panniculitis and subcutaneous SS [6, 7], while the presence of ischemic fat necrosis makes the diagnosis of EN and subcutaneous SS less likely [7, 8]. In addition, EN typically show septal panniculitis in the absence of vasculitis, which was inconsistent with our findings [8]. Ultimately, the diagnosis of infective panniculitis in our case is confirmed by the detection of *B. pseudomallei* in tissue aerobic culture.

Upon receiving the culture result, we conducted a re-interview with the patient. It was revealed that she resides in Mukdahan, a province in northeastern Thailand known to be highly endemic for melioidosis [9, 10]. Additionally, her hobby of caring for cacti frequently exposed her to soil and involved multiple instances of cactus pricks on her palms and forearms, increasing her susceptibility to inoculation. The thicker stratum corneum in the palms could explain why infection-induced panniculitis only developed in the forearm in our case. Since she has been pricked by cacti on a regular basis, she was unable to reliably recall when she was last pricked on the site with panniculitis. She denied any prior history of cellulitis or any cutaneous lesion prior to this incident. A similar case of nodular lymphangitis caused by nocardiosis following mild trauma from a cactus has been previously reported [11]. 

Skin and soft tissue infections are prevalent among immunocompetent patients as well as in immunocompromised patients, especially with hematologic malignancies [12]. Pseudomonas aeruginosa is a frequent agent that typically manifests as ecthyma gangrenosum, in addition to common bacterial skin infections such as streptococci and staphylococci [12]. Additionally, it is important to consider the possibility of fungi and rapid-growing nontuberculous mycobacteria infections [12]. Infective panniculitis is also frequently observed in immunocompromised patients, and histopathologic examinations frequently reveal a mixed septal-lobular panniculitis with neutrophilic infiltration, hemorrhage, and fat necrosis [6, 13]. It may result from direct inoculation into the subcutaneous tissue, hematogenous spreading to the subcutaneous tissue, or direct extension from the underlying source of infection [6]. Infectious panniculitis can be caused by a variety of organisms, such as bacteria, viruses, mycobacteria, and fungi [14]. Given that her skin lesion was solitary, preceding her sepsis, and considering her risk of *B. pseudomallei* inoculation, we hypothesize that her melioidosis originated from the skin. This hypothesis is supported by the fact that *B. pseudomallei* was detected only in the incisional biopsy specimen and not in the hemocultures, consistent with the rarity of bacteremia in primary cutaneous melioidosis [1]. 

Melioidosis is recognized to be linked to comorbidities that impair the immune system, particularly diabetes [15]. Patients with CML are at an elevated risk of community-acquired infection; [16] however, our patient was undergoing TKI treatment for CML at the time of lesion onset, which may have further increased her susceptibility to infection. While ponatinib may induce neutropenia and severe infection in long-term data [17, 18], however, the overall risk of infection remains generally low [19]. The extent to which CML and TKI therapy predisposes patients to melioidosis warrants further investigation.

The treatment for melioidosis-induced skin abscess involves an initial intensive therapy lasting a minimum of 10–14 days, using antibiotics such as ceftazidime, meropenem, or imipenem [20]. This is followed by an eradication phase of at least three months with trimethoprim–sulfamethoxazole [20]. In our case, meropenem was prescribed for the majority of her intensive therapy due to her critical condition during the early febrile neutropenia phase. The eradication phase was completed with oral trimethoprim–sulfamethoxazole, without the development of cytopenia or any drug interactions. Nevertheless, she was able to recover, and her skin lesion was healed without the need of surgical intervention.

## Conclusion

Melioidosis is an emerging infectious disease with various cutaneous manifestations. To the best of our knowledge, we present the first case of cutaneous melioidosis manifesting as infective panniculitis, highlighting the necessity of considering melioidosis as a potential infectious agent in patients who present with panniculitis with immunosuppression risk, a history of minor trauma, and live in melioidosis-endemic regions.

## Electronic supplementary material

Below is the link to the electronic supplementary material.

Supplementary Material 1

Supplementary Material 2

Supplementary Material 3

## Data Availability

No datasets were generated or analysed during the current study.

## Abbreviations

CML: Chronic myeloid leukemia
TKI: Tyrosine kinase inhibitor
ASCT: Allogenic stem cell transplantation
MIC: Minimum inhibitory concentration

## References

[1] Schwartzman G, Reddy SA, Berg SH, Currie BJ, Saavedra AP. Cutaneous melioidosis: an updated review and primer for the dermatologist. J Am Acad Dermatol. 2023;89:1201–8. 37582471 10.1016/j.jaad.2023.07.1032

[2] Wiersinga WJ, Virk HS, Torres AG, Currie BJ, Peacock SJ, Dance DAB, et al. Melioidosis Nat Reviews Disease Primers. 2018;4:17107. 29388572 10.1038/nrdp.2017.107 PMC6456913

[3] Vithoosan S, Thanushah B, Piranavan P, Gamlaksha D, Karunatilake H, Jayanaga A. A rare case of sweet syndrome secondary to melioidosis. BMC Dermatol. 2019;19:16. 31787085 10.1186/s12895-019-0096-2 PMC6886164

[4] Choonhakarn C, Jirarattanapochai K. Cutaneous polyarteritis Nodosa: a report of a case associated with melioidosis (Burkholderia pseudomallei). Int J Dermatol. 1998;37:433–6. 9646129 10.1046/j.1365-4362.1998.00403.x

[5] Jorgensen JH, Hindler JF. New consensus guidelines from the clinical and laboratory standards Institute for antimicrobial susceptibility testing of infrequently isolated or fastidious bacteria. Clin Infect Dis. 2007;44:280–6. 17173232 10.1086/510431

[6] Morrison LK, Rapini R, Willison CB, Tyring S. Infection and panniculitis. Dermatol Ther. 2010;23:328–40. 20666820 10.1111/j.1529-8019.2010.01333.x

[7] Guhl G, García-Díez A. Subcutaneous sweet syndrome. Dermatol Clin. 2008;26:541–51. viii-ix. 18793988 10.1016/j.det.2008.06.003

[8] Limtong P, Suchonwanit P, Chanprapaph K, Rutnin S. Clinicopathological characteristics related to etiologies of erythema nodosum: A 10-Year retrospective study. Clin Cosmet Investig Dermatol. 2021;14:1819–29. 34876828 10.2147/CCID.S343351 PMC8643131

[9] Hinjoy S, Hantrakun V, Kongyu S, Kaewrakmuk J, Wangrangsimakul T, Jitsuronk S, et al. Melioidosis in Thailand: present and future. Trop Med Infect Dis. 2018;3:38. 29725623 10.3390/tropicalmed3020038 PMC5928800

[10] Chantratita N, Phunpang R, Yarasai A, Dulsuk A, Yimthin T, Onofrey LA et al. Characteristics and one year outcomes of melioidosis patients in Northeastern Thailand: A prospective, multicenter cohort study. Lancet Reg Health Southeast Asia 2023;9. 10.1016/j.lansea.2022.100118 PMC9788505 36570973

[11] De Cantón J, Cabanillas Navarro I, Quevedo Soriano S. Lois Martínez N. Nodular lymphangitis due to nocardiosis. BMJ Case Rep 2022;15. 10.1136/bcr-2022-252941 PMC9806021 36581362

[12] Ungaro R, Mikulska M. The skin and soft tissue infections in hematological patients. Curr Opin Infect Dis. 2020;33:101–9. 32022740 10.1097/QCO.0000000000000632 PMC9930892

[13] Patterson JW, Brown PC, Broecker AH. Infection-induced panniculitis. J Cutan Pathol. 1989;16:183–93. 2794161 10.1111/j.1600-0560.1989.tb00038.x

[14] Delgado-Jimenez Y, Fraga J, García-Díez A. Infective panniculitis. Dermatol Clin. 2008;26:471–.– 80, vi. 18793979 10.1016/j.det.2008.05.005

[15] Fertitta L, Monsel G, Torresi J, Caumes E. Cutaneous melioidosis: a review of the literature. Int J Dermatol. 2019;58:221–7. 30132827 10.1111/ijd.14167

[16] Titmarsh GJ, McMullin MF, McShane CM, Clarke M, Engels EA, Anderson LA. Community-acquired infections and their association with myeloid malignancies. Cancer Epidemiol. 2014;38:56–61. 24275260 10.1016/j.canep.2013.10.009 PMC3943929

[17] Cortes JE, Kim D-W, Pinilla-Ibarz J, le Coutre PD, Paquette R, Chuah C, et al. Ponatinib efficacy and safety in Philadelphia chromosome–positive leukemia: final 5-year results of the phase 2 PACE trial. Blood. 2018;132:393–404. 29567798 10.1182/blood-2016-09-739086 PMC6071555

[18] Ruiz-Camps I, Aguilar-Company J. Risk of infection associated with targeted therapies for solid organ and hematological malignancies. Ther Adv Infect Dis. 2021;8:2049936121989548. 33680453 10.1177/2049936121989548 PMC7897815

[19] Kin A, Schiffer CA. Infectious complications of tyrosine kinase inhibitors in hematological malignancies. Infect Dis Clin North Am. 2020;34:245–56. 32444010 10.1016/j.idc.2020.02.008

[20] Meumann EM, Limmathurotsakul D, Dunachie SJ, Wiersinga WJ, Currie BJ. Burkholderia Pseudomallei and melioidosis. Nat Rev Microbiol. 2024;22:155–69. 37794173 10.1038/s41579-023-00972-5

