# Supplementary figures and images for: A rare case of cutaneous melioidosis manifesting as infective panniculitis: a case report
Source: BMC Infect Dis. 2025 Apr 18;25:559. doi: 10.1186/s12879-025-10939-x (PMC12008837; doi:10.1186/s12879-025-10939-x)

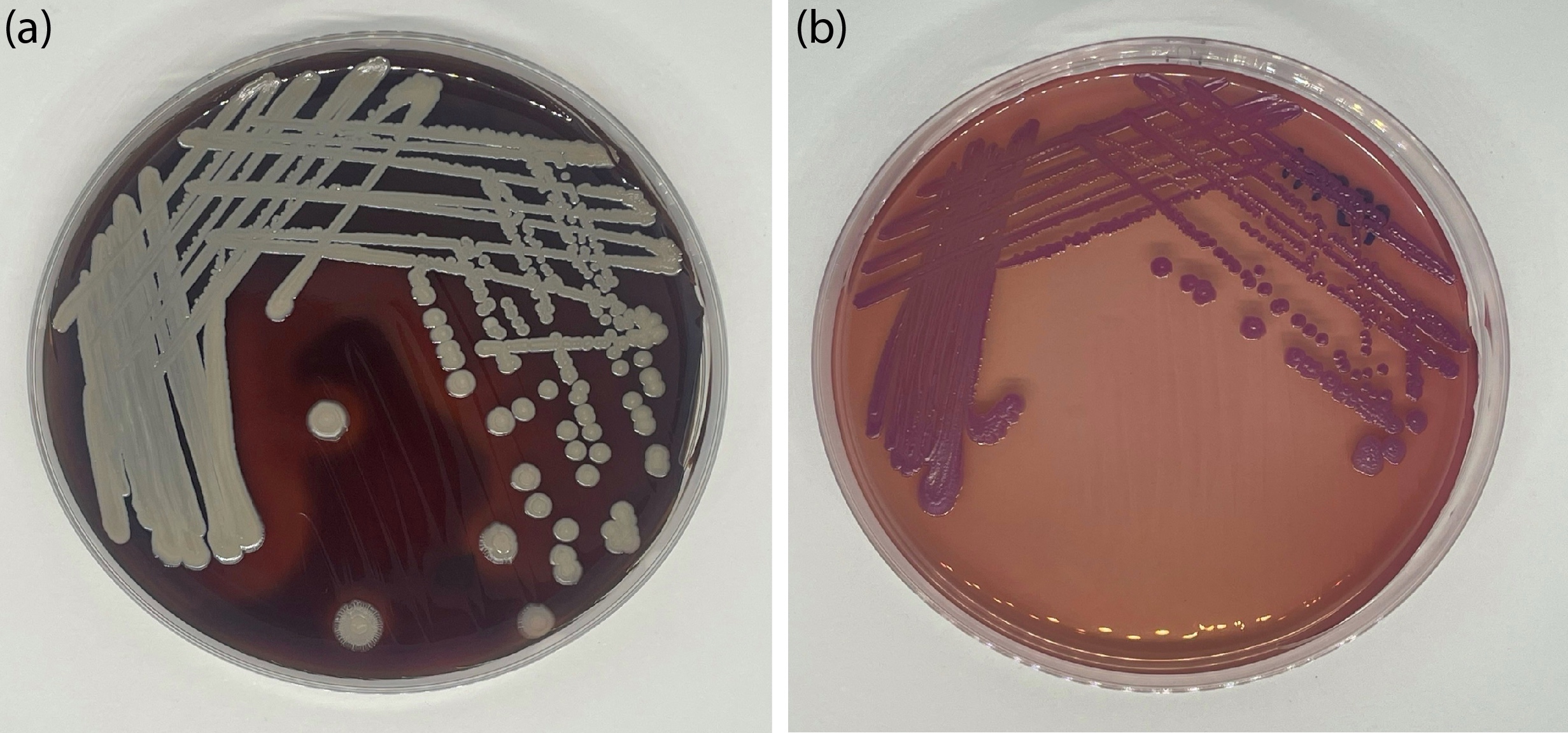

Supplement: Supplementary file 1 — Supplementary Material 1 [file 12879_2025_10939_MOESM1_ESM.jpg]

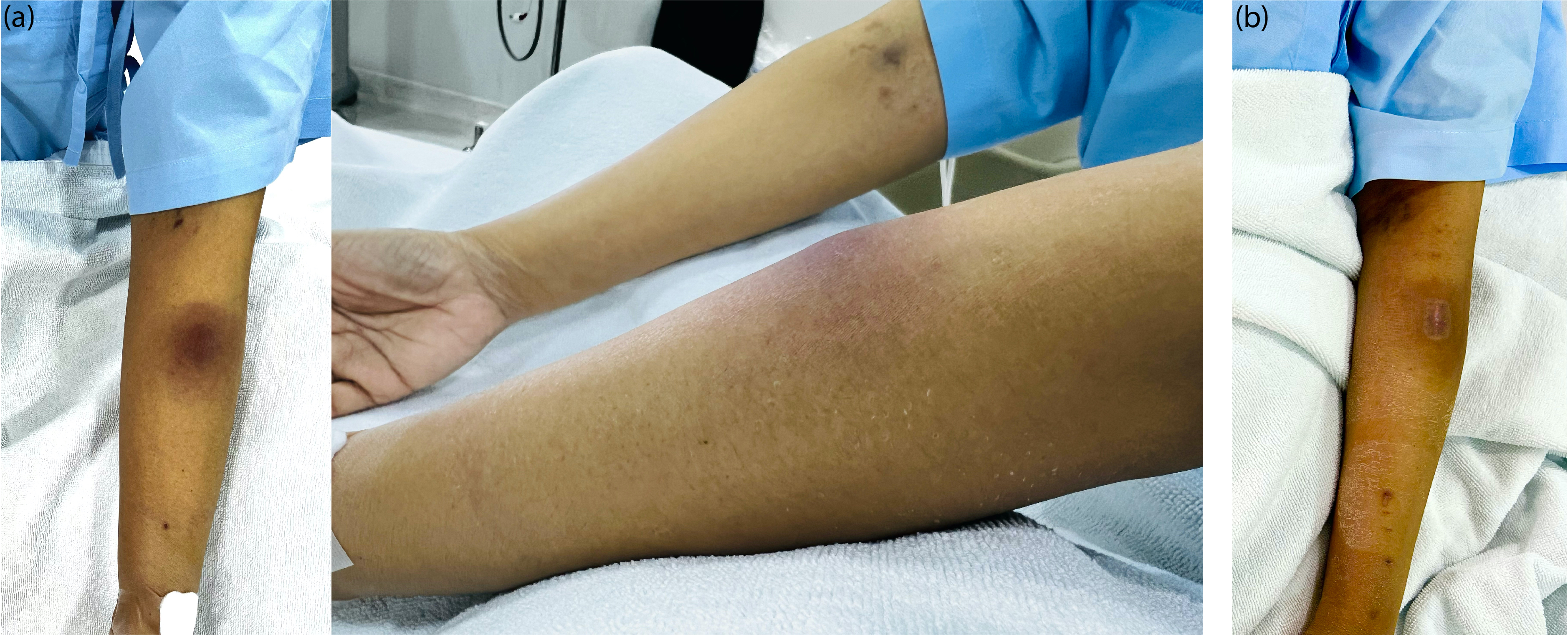

Supplement: Supplementary file 2 — Supplementary Material 2 [file 12879_2025_10939_MOESM2_ESM.jpg]

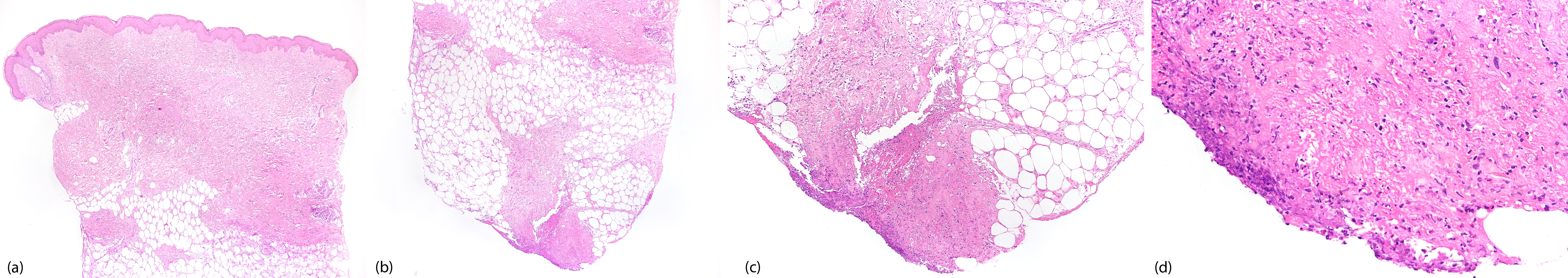

Supplement: Supplementary file 3 — Supplementary Material 3 [file 12879_2025_10939_MOESM3_ESM.jpg]
